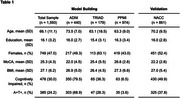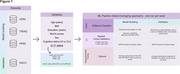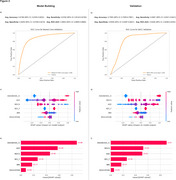# Machine Learning Approach for Predicting Amyloid and Tau Positivity in Alzheimer's Disease Using Clinically Accessible Features

**DOI:** 10.1002/alz70856_105937

**Published:** 2026-01-08

**Authors:** Daniel Arnold, Luiza Santos Machado, Nesrine Rahmouni, Joseph Therriault, Stijn Servaes, Jenna Stevenson, Arthur C. Macedo, Artur Francisco Schumacher‐Schuh, Christian Mattjie, Firoza Z Lussier, Mira Chamoun, Gleb Bezgin, Andrea L. Benedet, Tharick A Pascoal, Rodrigo C. Barros, Marco De Bastiani, Pedro Rosa‐Neto, Eduardo R. Zimmer, Wyllians Vendramini Borelli, Alzheimer's Disease Neuroimaging Initiative

**Affiliations:** ^1^ Universidade Federal do Rio Grande do Sul, Porto Alegre, Rio Grande do Sul, Brazil; ^2^ University of Gothenburg, Gothenburg, VG, Sweden; ^3^ Montreal Neurological Institute, Montreal, QC, Canada; ^4^ McGill University, Montreal, QC, Canada; ^5^ Hospital de Clínicas de Porto Alegre, Porto Alegre, RS, Brazil; ^6^ PUCRS, Porto Alegre, Rio Grande do Sul, Brazil; ^7^ University of Pittsburgh, Pittsburgh, PA, USA; ^8^ Department of Psychiatry and Neurochemistry, Institute of Neuroscience and Physiology, The Sahlgrenska Academy, University of Gothenburg, Mölndal, Sweden; ^9^ Brain Institute of Rio Grande do Sul (InsCer), PUCRS, Porto Alegre, Rio Grande do Sul, Brazil; ^10^ McGill Centre for Studies in Aging, Montreal, QC, Canada; ^11^ Centro de Memória, Hospital Moinhos de Vento, Porto Alegre, RS, Brazil; ^12^ Clinical Hospital of Porto Alegre, Porto Alegre, Rio Grande do Sul, Brazil; ^13^ Laboratory of Neuro Imaging (LONI), University of Southern California, Los Angeles, CA, USA

## Abstract

**Background:**

Prediction of Alzheimer's disease (AD) biomarkers can improve public health strategies, especially if achieved with easily collectable data in a single consultation. Machine learning (ML) offers versatile tools for clinical and research applications. This study investigated a ML model's ability to predict amyloid and tau positivity using easily obtainable features.

**Method:**

Individuals with amyloid and tau status were selected from ADNI, TRIAD, and PPMI datasets (Model Building) and from the NACC dataset (Validation). Shared clinical features included age, sex, education, clinical diagnosis, MoCA scores, and BMI. Amyloid positivity was defined by amyloid‐PET (PIB‐PET, FBB‐PET, or AZD4694‐PET) or CSF AB42, and tau positivity by Tau‐PET (MK6240‐PET, AV1451‐PET) or CSF *p*‐tau181. For NACC, positivity derived from fields AMYLPET and TAUPETAD. Data processing is summarized in Figure 1.

**Result:**

The Model Building sample included 1593 individuals (mean MoCA 25.3 ± 4.3). The Validation cohort included 861 individuals (mean MoCA 22.2 ± 2.8). The model achieved high performance for predicting amyloid and tau positivity, with mean AUCs of 0.89 and 0.83 for Model Building and Validation, respectively (Figure 2a, 2b). In Validation, high sensitivity (0.93) came at the expense of specificity (0.65), while Model Building showed balanced sensitivity and specificity (0.81 and 0.83). Higher age, lower MoCA, cognitive impairment, female sex, and lower BMI increased the probability of positivity (Figure 2c, 2d). Cognitive impairment was the most impactful feature in both Model Building and Validation datasets, followed by MoCA and age (Figure 2e, 2f).

**Conclusion:**

Predicting AD biomarkers using ML and readily collectable features is feasible and accurate. The model's high sensitivity indicates a potential research utility in clinical trials for population screening to minimize false negatives. Future efforts should enhance generalizability, explore additional features, and prioritize real‐world validation.